# Prevalence and Predictors of Hemorrhagic Foci on Long-term Follow-up MRI of Recent Single Subcortical Infarcts

**DOI:** 10.1007/s12975-023-01224-7

**Published:** 2023-12-14

**Authors:** Shuai Jiang, Wen-Zuo Shang, Jing-Yu Cui, Yu-Ying Yan, Tang Yang, Yi Hu, Le Cao, Xun Yue, Ruo-Su Pan, Chen Ye, Jia-Yu Sun, Bo Wu

**Affiliations:** 1 Department of Neurology, West China Hospital, Sichuan University, No. 37, Guo Xue Xiang, Chengdu, 610041 China; 2 Department of Radiology, West China Hospital, Sichuan University, Guo Xue Xiang 37, Chengdu, 610041 China

**Keywords:** Lacune, Hemorrhagic foci, Lenticulostriate Artery, Recent Single Subcortical Infarcts, Susceptibility-weighted Imaging

## Abstract

Hemorrhagic foci surrounding the lacune in the long-term evolution of recent single subcortical infarcts (RSSIs) remains largely unexplored. We aimed to determine the prevalence, characteristics, and predictors of hemorrhagic foci in patients with RSSI. From a prospective, longitudinal study of RSSIs, we recruited patients who underwent multimodal MRI assessments both at baseline and approximately one year after the stroke onset. Hemorrhagic foci were identified using susceptibility-weighted imaging (SWI). Among 101 patients with RSSI, nearly half (n = 45, 44.6%) had hemorrhagic foci within the index RSSI lesions on follow-up SWI. RSSIs with hemorrhagic foci formation were associated with a longer time to follow-up imaging (median 449 versus 401 days, P = 0.005) and higher likelihood of being located in the anterior circulation compared to those without hemorrhagic foci (88.9% versus 64.3%, P = 0.003). Hemorrhagic foci were also associated with larger lesion size (P < 0.001), a higher proportion of cavitation formation (P = 0.003), higher baseline NIHSS scores (P = 0.004), and poorer functional outcomes (P = 0.001). In the subset of RSSIs in the lenticulostriate artery (LSA) territory, after adjustment for covariates, larger initial lesion volume (OR 1.80, 95% CI 1.13–2.87; P = 0.014) and greater decreases in LSA total length (OR 0.59, 95% CI 0.36–0.96; P = 0.035) were independently associated with hemorrhagic foci formation. The extent of ischemia in the initial infarct is predictive of the presence of hemorrhagic residues. Our findings contribute to the current understanding of the mechanisms underlying the evolution of RSSIs.

**Supplementary Information:**

The online version contains supplementary material available at 10.1007/s12975-023-01224-7.

## Introduction

Recent single subcortical infarcts (RSSIs) are regarded as one of the acute radiological manifestations of cerebral small vessel disease (CSVD) on MRI, accompanied by relevant clinical symptoms [1, 2]. Previous studies have demonstrated that RSSIs exhibit diverse outcomes, ranging from cavitation to a lacune formation, non-cavitated white matter hyperintensities (WMH), or even complete disappearance on follow-up imaging [3–8]. The updated Standards for Reporting Vascular Changes on Neuroimaging 2 (STRIVE-2) also describe how RSSIs evolution can reveal the presence of a hemosiderin (T2*-hypointense) rim surrounding the lacune or a small hemosiderin (T2*-hypointense) smudge [2]. However, the hemorrhagic evolutions of RSSIs during long-term follow-up have received little attention in radiological investigations, and the clinical and pathophysiological significance of this process is largely unexplored.

On the contrary, hemorrhagic features of chronic lacunes have been the subject of considerable pathological investigation. Miller Fisher’s meticulous postmortem work, conducted nearly a half century ago, revealed scattered hemosiderin-filled macrophages in the vicinity of the cavities, suggesting the occurrence of small hemorrhagic extravasations through the responsible disorganized arteries [9–11]. Furthermore, in the classical neuropathological classification of lacunes by Poirier and Derouesne, type II lacunes were defined as cavities filled with numerous hemosiderin-laden macrophages [12, 13]. Though commonly assumed to represent old small hemorrhages, it is equally plausible that some of them might actually represent previous hemorrhagic exudate of lacunar infarcts.

Susceptibility-weighted imaging (SWI) represents an advance in T2*-weighted brain magnetic resonance imaging (MRI) that enables the detection of millimeter-sized paramagnetic blood products, including hemosiderin, within the brain parenchyma [14]. It has been widely utilized to identify hemorrhagic features in CSVD. The recent development of intracranial vessel wall imaging (VWI) now allows for enhanced visualization of lenticulostriate arteries (LSAs) [15, 16]. Since LSA occlusion is considered the main cause of RSSIs in the middle cerebral artery (MCA) territory, [11] tracking changes in LSA morphology over time may provide valuable insights into the long-term fate of RSSIs, and help better characterize hemorrhagic evolution in the LSA territory.

We hypothesized that a larger reduction in LSA length on longitudinal MRI may indicate more substantial ischemic damage and be linked to the presence of hemorrhagic foci within the RSSIs. Here, we investigate the prevalence and characteristics of these hemorrhagic foci observed on follow-up SWI, and characterize their associations with clinical features, infarct size, CSVD neuroimaging markers, and LSA morphology in patients with RSSI.

## Methods

### Patients

Patients were drawn from the prospective RSSI study conducted by the Department of Neurology at West China Hospital between July 2018 to July 2022. All patients gave informed consent to participate in this research project, [16–18] which was approved by the local medical ethics committee (2,018,521). In the present analysis, we included RSSI patients with (1) a first-ever RSSI in penetrating arterial territory with relevant clinical symptoms identified on diffusion-weighted imaging (DWI) and (2) a follow-up MRI approximately one year after the index stroke. We refrained from establishing specific upper size limits, such as the commonly used 15 or 20 mm for RSSI, considering that DWI may overestimate infarct size due to edematous swelling of infarction tissue and because the use of an absolute size cutoff remains controversial [19, 20].

**Fig. 1:**
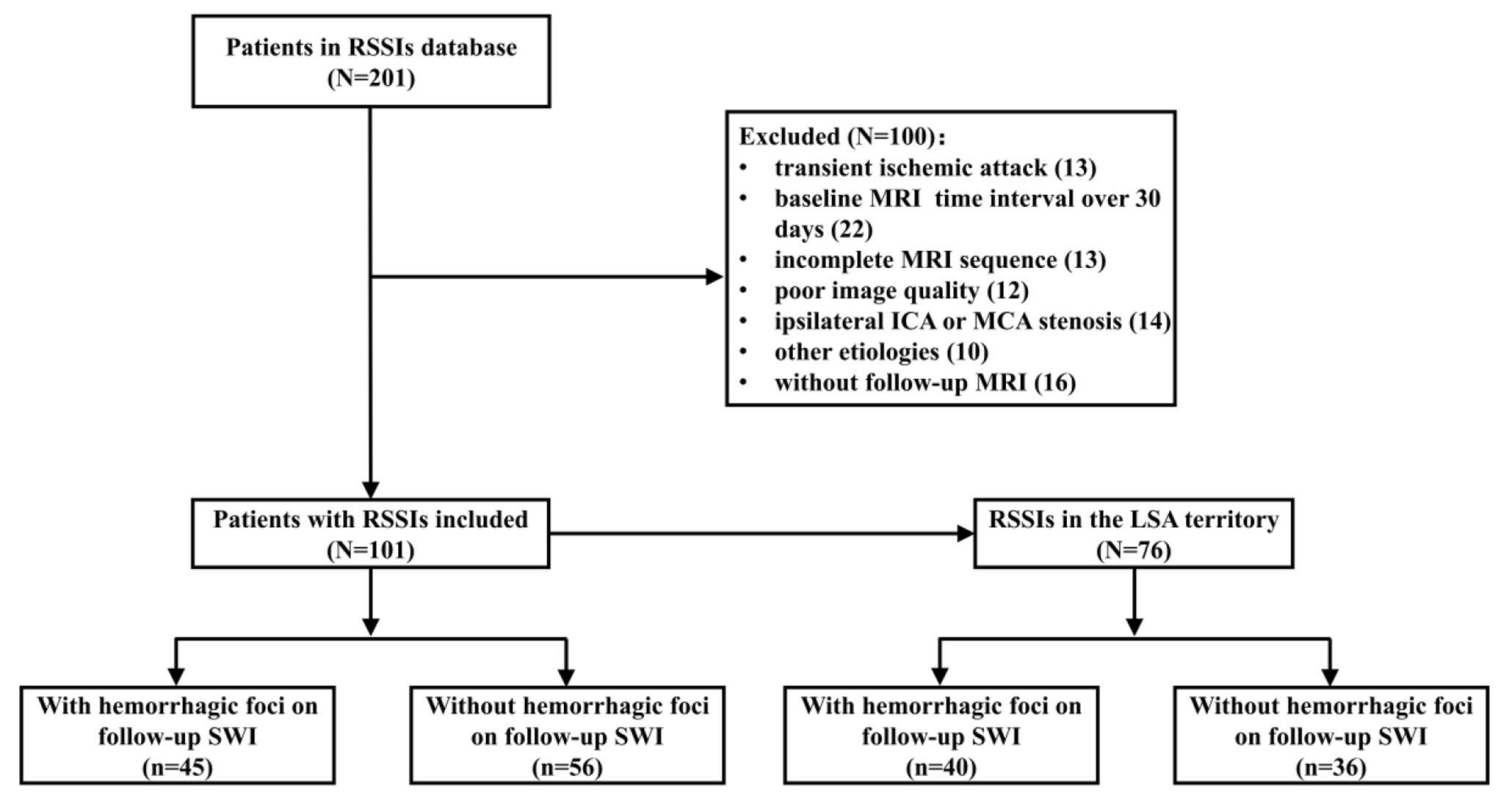
Flowchart of patient selection. Abbreviations: RSSIs = recent single subcortical infarcts; DWI = diffusion-weighted imaging; MCA = middle cerebral artery; ICA = internal carotid artery; LSA = lenticulostriate artery; SWI = susceptibility-weighted imaging

Patients with evidence of cardioembolism, coexisting ≥ 50% stenosis at the ipsilateral intracranial internal carotid artery or relevant extracranial arteries detected by computed tomography angiography or magnetic resonance angiography (MRA), as well as those with non-atherosclerotic vasculopathies (e.g., vasculitis, moyamoya disease, dissection), were excluded from the study. All patients underwent a thorough neurological examination and cerebrovascular workup, including routine blood tests, 24-hour electrocardiographic or Holter monitoring, transthoracic echocardiography, and brain MRI at baseline. The flowchart of the patient selection is shown in Fig. 1.

Demographics, vascular risk factors, and medication use were also recorded. Stroke severity was determined using the National Institutes of Health Stroke Scale (NIHSS) at the time of admission. Functional outcome was assessed by trained staff using the modified Rankin scale (mRS) at follow-up. A poor outcome was defined as an mRS ≥ 2 at the follow-up visit.

### MRI Protocols

All MRI scans (baseline and follow-up) were obtained on a research-dedicated 3.0-tesla MRI (MAGNETOM Trio, Siemens, Erlangen, Germany) with a 32-channel head coil. The standardized protocol included T1-weighted, T2-weighted, fluid-attenuated inversion recovery (FLAIR), DWI, three-dimensional time-of-flight MRA (3D TOF-MRA), SWI, and high-resolution whole-brain VWI sequences. Detailed sequence parameters have previously been described (online supplemental table S1) [17, 18].

### Image Analysis

All images were reviewed and analyzed using a commercially available software package (Osirix MD, Pixmeo SARL). An experienced stroke neurologist (S.J.) and an experienced neuroradiologist (J.S.), both blinded to all clinical data, independently viewed a random selection of 20 anonymized scans and identified the presence of hemorrhagic foci. To calculate intra-rater reliability, S.J. repeated the assessment after an interval of 2 months. In cases of disagreement, a third senior stroke neurologist (B.W.) was invited to reach a consensus.

### Infarct Size and RSSI Evolution

RSSIs were defined as round or ovoid DWI hyperintense lesions confirmed by corresponding low signal changes on the apparent diffusion coefficient (ADC) map located in the basal ganglia, internal capsule, thalamus, corona radiata, or brainstem. In cases where the extension of the infarct affected both of these regions, we assigned the most represented location. RSSIs were manually segmented on the DWI using Osirix software and then co-registered with the follow-up images to confirm the lesion of observation. The initial maximal axial lesion diameter and lesion volume were calculated based on DWI sequences for the RSSI and on follow-up T1-weighted images for the corresponding lacune of the index RSSI. On follow-up MRI scans, the evolution of the index RSSI was classified as either cavitation, WMH, or disappearance [3, 21].

The presence of cavitation was defined as a cavitated lesion with consistent cerebrospinal fluid signal intensity or near equivalent intensity (hypointensity) surrounded with or without a high signal circle on the FLAIR sequence. RSSIs that developed into hyperintensity lesions as WMH or disappeared on follow-up FLAIR images were classified as non-cavitation [1].

**Fig. 2:**
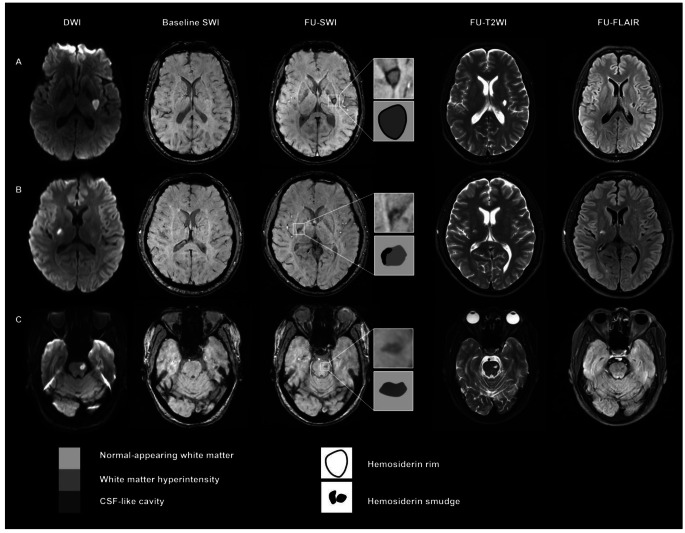
Representative cases of hemorrhagic foci on follow-up MRI. Hemorrhagic foci were evaluated within the designated squares and have been magnified to provide detailed visualization on susceptibility-weighted imaging (SWI). Notably, the initial SWI scans did not reveal any evidence of hemorrhagic remnants. The schematic representation of hemorrhagic foci on SWI was depicted in the corresponding squares under the magnifications. (**A**) A left lenticular nucleus recent single subcortical infarct (RSSI) was observed on diffusion-weighted imaging (DWI), and a corresponding lacune was visible on follow-up FLAIR. The index lacune exhibited a characteristic dark hemosiderin rim on follow-up SWI, with an additional irregular low-signal rim appearance at the edges of the lacune observed on follow-up T2-weighted imaging (T2WI). (**B**) A right capsular infarct seen on DWI showed corresponding white matter hyperintensity (WMH) on follow-up FLAIR. A lacune was visible in the upper slice (not shown). Patch-like hemosiderin deposits with marked lower signal intensity compared to the surrounding tissue (WMH) were visible on follow-up SWI. No low signal intensity was identified in the corresponding region on T2WI. (**C**) A left pontine infarct was identified on DWI, and a corresponding lacune was observed on follow-up, but no low-signal intensity was visible within the lacune areas on follow-up SWI and T2WI.

Hemorrhagic foci were identified as a low-signal rim or, in some cases, prominent dark patch surrounding or within the lacune of the index RSSI on SWI, indicative of the presence of hemosiderin deposits. When located within the cavity, low-signal dots exhibited significantly lower signal intensity compared to the surrounding cystic component of a lacune (Fig. 2).

### LSA Morphology and CSVD Markers

**Fig. 3:**
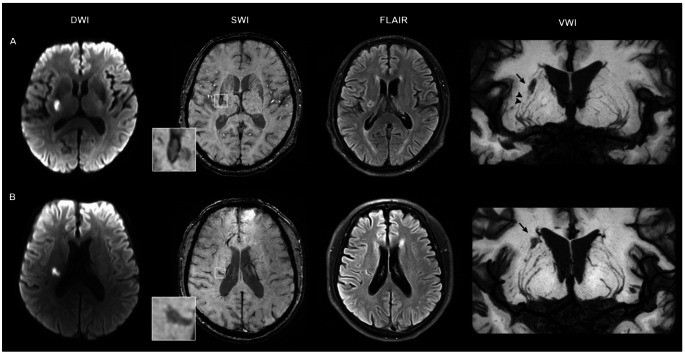
Correlation between changes in LSA characteristics and hemorrhagic foci. (**A**) A recent single subcortical infarct (RSSI) showed cavitated evolution on follow-up fluid-attenuated inversion recovery (FLAIR), accompanied by a typical hemosiderin rim surrounding the cavity (magnified view in the square). Coronal minimum intensity projection (MinIP) of vessel wall imaging (VWI) revealed shorter lengths of the right lenticulostriate arteries (LSAs) (arrowheads) compared to the left side. The black arrow indicated the index cavity on VWI. (**B**) A RSSI evolved into a cavity on follow-up, but no hemosiderin deposits were observed on follow-up SWI (magnified view in the square). Coronal MinIP revealed relatively symmetrical LSAs on both the right and left sides. The black arrow indicated the index cavity on VWI.

LSA images were generated using coronal minimum intensity projection (MinIP) on VWI (Fig. 3). The morphological characteristics of visible LSAs including the number of stems and branches, total length and distance were quantitatively analyzed. Changes in LSA stems, branches, total length and distance were expressed as differences (follow-up - baseline). Details of LSA morphometry have been published elsewhere [16, 17].

Baseline radiologic markers of CSVD, including lacunes of presumed vascular origin (separate from the index lesion), perivascular spaces (PVS), cerebral microbleeds (CMBs), and WMH were identified according to the STRIVE criteria [1]. Deep and periventricular WMHs were both coded according to the Fazekas scale from 0 to 3, where a score ≥ 2 was regarded as extensive WMH. PVS was evaluated using a validated semiquantitative scale ranging from 0 to 4 in the basal ganglia region, with a grade of 2–4 categorized as moderate to extensive.

### Statistical Analysis

All quantitative data were reported as mean and standard deviation (SD) or median with interquartile range (IQR), while qualitative data were summarized as count (percentage). Intra-rater and inter-rater agreements for the presence of hemorrhagic foci were determined using the Cohen kappa coefficient and graded according to published criteria [22]. For categorical variables, appropriate tests such as Chi-square or Fisher exact test were utilized, while the t-test (normally distributed variables) or Mann-Whitney U test (non-normally distributed data) were employed to compare continuous variables between the two groups. Multivariable logistic regression models were utilized to identify independent predictors of the presence of hemorrhagic foci on follow-up MRI, incorporating variables with a P-value < 0.1 from univariate analysis. Collinearity was tested using the variance inflation factor (< 10 for all variables). Statistical analysis and plotting were conducted with the R software package (version 4.2.03). Raincloud plots were generated using the ggrain package. Two-tailed values of P < 0.05 were considered statistically significant.

## Results

### Patient Characteristics

From July 2018 to July 2022, 201 patients were recruited in the original RSSI study and 101 patients meeting the eligibility criteria for the present study were enrolled. Details on recruitment and study flow are shown in Fig. 1. The overall mean age of study participants was 54.11 ± 9.90 years, and 81 (80.2%) of them were male. Median time from stroke symptom onset to follow-up imaging was 425 (377–522) days. RSSIs in these patients were located most commonly in the corona radiata (47.5%), with other locations including the basal ganglia or internal capsule (27.7%), brainstem (6.0%), and thalamus (18.8%). At the time of follow-up imaging, 79 patients (78.2%) showed cavitation of the RSSI lesions on FLAIR, 16 (15.8%) index lesions resembled a non-cavitated WMH, and 6 (6.0%) index lesions had disappeared during follow-up. Other clinical and neuroimaging characteristics of the study cohort are presented in Table 1.

**Table 1:** Demographic, clinical data and imaging characteristics of the total study cohort and patients with or without hemorrhagic foci on follow-up MRI

Characteristics	Total cohort (n = 101)	Hemorrhagic foci (n = 45)	No hemorrhagic foci (n = 56)	*p* Value
**Demographics**				
Male, n (%)	81 (80.2)	39 (86.7)	42 (75.0)	0.144
Age, y (mean ± SD)	54.11 ± 9.90	54.22 ± 9.87	54.02 ± 10.01	0.918
**Risk factors, n (%)**				
Hypertension	59 (58.4)	23 (51.1)	36 (64.3)	0.182
Diabetes	34 (33.7)	19 (42.2)	15 (26.8)	0.103
Hyperlipidemia	35 (34.7)	19 (42.2)	16 (28.6)	0.152
Current smoking	50 (49.5)	20 (44.4)	30 (53.6)	0.362
**Clinical data, median (IQR)**				
Baseline NIHSS	3 (1–4)	3 (2–8)	2 (1–3.5)	0.004
Baseline mRS	2 (1–4)	3 (1.5–4)	2 (1–3)	0.023
Follow-up mRS	1 (1–2)	1 (1–2)	1 (0–1)	0.004
Follow-up mRS ≥ 2, n (%)	28 (27.7)	20 (44.4)	8 (14.3)	0.001
Onset to baseline MRI time, days	6 (3–8)	5 (4–7)	6 (3–11.5)	0.081
Onset to follow-up MRI time, days	425 (377–522)	449 (400–603)	401 (371.5-464.5)	0.005
**Medication, n (%)**				
Dual antiplatelet therapy	48 (47.5)	24 (53.3)	24 (42.9)	0.295
**RSSI location, n (%)**				0.003
Basal ganglia/internal capsule	28 (27.7)	18 (40.0)	10 (17.9)	
Corona radiata	48 (47.5)	22 (48.9)	26 (46.4)	
Thalamus	19 (18.8)	5 (11.1)	14 (25.0)	
Brainstem	6 (6.0)	0 (0.0)	6 (10.7)	
**Infarct Dimensions, median (IQR)**				
DWI lesion axial diameter, mm	14.1 (10.5–20.5)	18.6 (14.1–25.3)	11.6 (9.5–16.2)	< 0.001
DWI lesion volume, cm^3^	1.19 (0.38–2.80)	2.54 (1.37–5.79)	0.50 (0.26–1.43)	< 0.001
Follow-up T1 lacune axial diameter ^a^, mm	8.29 (6.50–10.80)	9.64 (7.69-12.00)	7.37 (5.31–8.63)	0.001
Follow-up T1 lacune volume, median ^a^, cm^3^	0.17 (0.09–0.36)	0.27 (0.13–0.57)	0.11 (0.05–0.19)	< 0.001
**CSVD markers, n (%)**				
Extensive perivascular WMH (Fazekas 3)	9 (8.9)	2 (4.4)	7 (12.5)	0.289
Extensive deep WMH (Fazekas 2–3)	15 (14.9)	4 (8.9)	11 (19.6)	0.131
Moderate–extensive BG-PVS	46 (45.5)	23 (51.1)	23 (41.1)	0.314
Lacunes (≥ l)	39 (38.6)	18 (40.0)	21 (37.5)	0.798
Cerebral microbleeds (≥ l)	45 (44.6)	21 (46.7)	24 (42.9)	0.702
**RSSI evolution, n (%)**				0.003
Cavitation, n (%)	79 (78.2)	41 (91.1)	38 (67.9)	
WMH, n (%)	16 (15.8)	4 (8.9)	12 (21.4)	
Disappearance, n (%)	6 (6.0)	0 (0.0)	6 (10.7)	

Data are presented as mean ± SD, median (IQR) or number (%)

^a^ Available in 79 patients, 16 lesions evolved into WMH and 6 lesions that disappeared on follow-up imaging were excluded

Abbreviations: BG-PVS = basal ganglia perivascular spaces; CSVD = cerebral small vessel disease; DWI = diffusion-weighted imaging; mRS = modified Rankin Scale; NIHSS = National Institutes of Health Stroke Scale; IQR = interquartile range; RSSI = recent single subcortical infarction; SD = standard deviation; T1 = T1 weighted image; WMH = white matter hyperintensities

### Comparisons of Clinical and Conventional Neuroimaging Characteristics with and without Hemorrhagic Foci Formation on Follow-up MRI

Almost half of the RSSI patients (n = 45, 44.6%) showed hemorrhagic foci within the index RSSI lesions on follow-up SWI. No hemorrhagic foci were observed from the initial MRI within the RSSI lesions. The intra-rater and inter-rater agreements for the presence of hemorrhagic foci were excellent (κ = 0.93, 95% CI: 0.80–0.99 and κ = 0.87, 95% CI: 0.69–0.99). We also found that the time interval between stroke onset and follow-up imaging was longer in patients with hemorrhagic foci (median 449 versus 401 days, P = 0.005). RSSIs with hemorrhagic foci formation were more likely to be located in the anterior circulation compared to those without hemorrhagic foci (40 [88.9%] versus 36 [64.3%]; P = 0.003).

In comparison to patients without hemorrhagic foci, patients with hemorrhagic foci had larger lesion axial diameter (median 18.6 versus 11.6 mm; P < 0.001) and lesion volume (median 2.54 versus 0.50 cm^3^; P < 0.001) on baseline DWI, as well as larger lacune axial diameter (median 9.64 versus 7.37 mm; P = 0.001) and lacune volume (median 0.27 versus 0.11 cm^3^; P < 0.001) at follow-up T1WI. Patients with hemorrhagic foci were also more likely to cavitate (91.1% versus 67.9%; P = 0.003) and had higher baseline NIHSS scores (3 [IQR 2–8] versus 2 [IQR 1–3.5]; P = 0.004), along with poorer functional outcomes (mRS ≥ 2) at follow-up (44.4% versus 14.3%; P = 0.001). We found that dual antiplatelet therapy had no effect on hemorrhagic foci formation (Table 1).

### LSA Characteristics between RSSIs in the LSA Territory with and without Hemorrhagic Foci Formation on Follow-up MRI

To evaluate the relationship between LSA characteristics and subsequent hemorrhagic foci (Fig. 3), we conducted further analysis on a subset of RSSIs in the LSA territory, excluding RSSIs located in the thalamus (n = 19) and brainstem (n = 6). Of the remaining 76 RSSIs, 40 (52.6%) were identified as hemorrhagic foci and 36 (47.4%) as non-hemorrhagic foci. Comparison results between these groups were similar to those for the whole cohort. Detailed analyses are shown in online supplemental table S2.

**Fig. 4:**
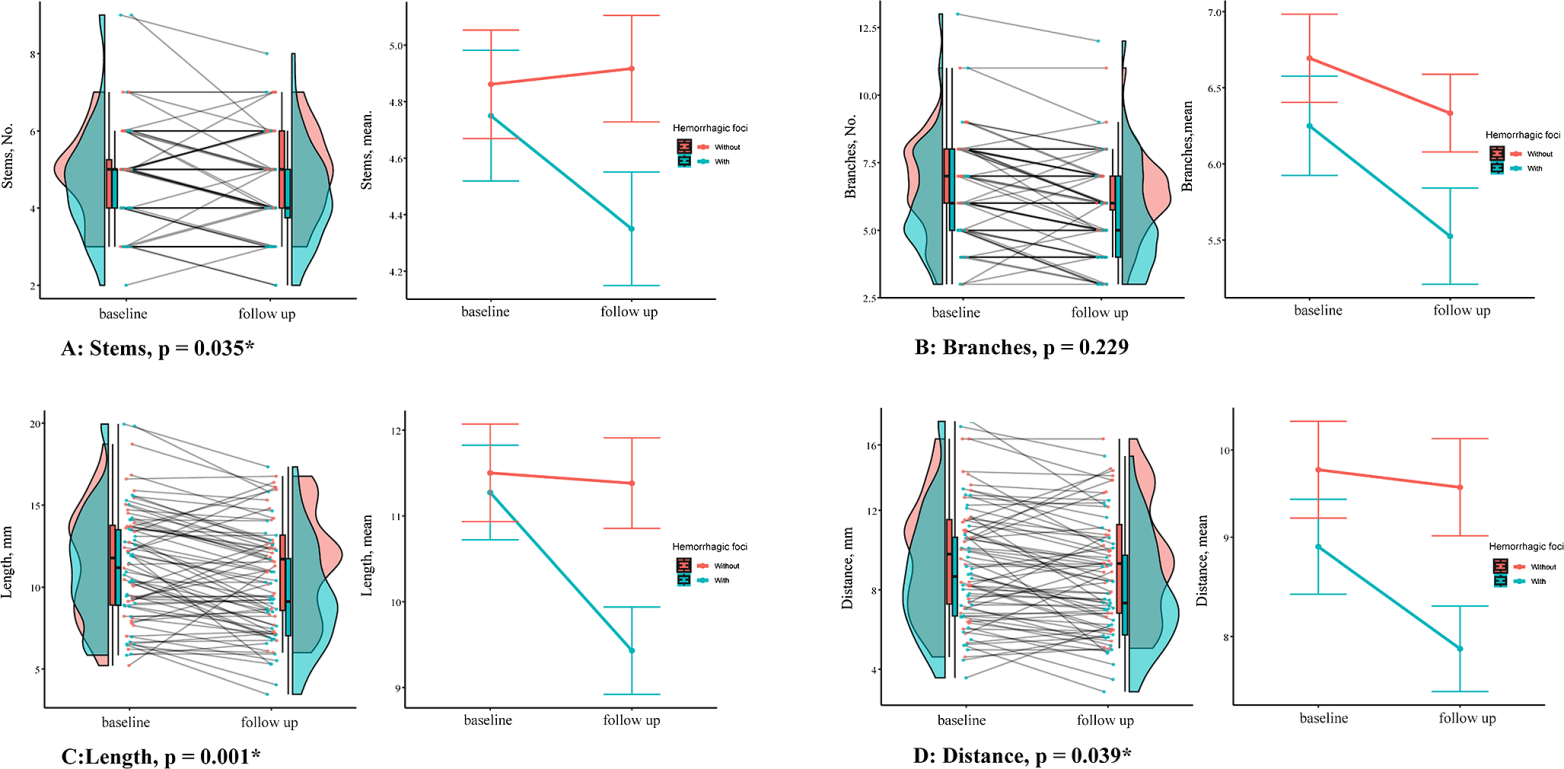
Changes in LSA characteristics during baseline and follow-up between patients with and without hemorrhagic foci. Patients with and without hemorrhagic foci formation at follow-up were labeled in blue and red, respectively. Greater decreases in LSA stems (P = 0.035), LSA total length (P = 0.001) and distance (P = 0.039) were observed in patients with versus without hemorrhagic foci formation during follow-up. The reduction in LSA branches was not significantly different between the two groups (P = 0.229)

The number of LSA stems, branches, total length and distance of LSAs at baseline were comparable between RSSIs with and without hemorrhagic foci. However, at follow-up, greater reduction in LSA stems (0 [IQR − 1–0] versus 0 [IQR − 0.5–1]; P = 0.035), total length (-18.44 ± 16.56 mm versus − 1.21 ± 23.73 mm, P = 0.001), and distance (-10.28 ± 15.53 mm versus − 1.77 ± 19.61 mm, P = 0.039) were observed in patients with hemorrhagic foci formation compared to those without. The reduction in LSA branches was not significantly different between the two groups (-1 [IQR − 1.5–0] versus 0 [IQR − 1–0]; P = 0.229) (Table 2; Fig. 4).

**Table 2:** Changes in LSA characteristics between RSSIs in the LSA territory with and without hemorrhagic foci

Characteristics	Hemorrhagic foci (n = 40)	No hemorrhagic foci (n = 36)	*p* Value
**LSA characteristics**			
Baseline number of LSA stems, median (IQR)	5 (4–5)	5 (4-5.5)	0.418
Follow-up number of LSA stems, median (IQR)	4 (3.5-5)	5 (4–6)	0.034
LSA stems change, median (IQR)	0 (-1-0)	0 (-0.5-1)	0.035
Baseline Number of LSA branches, median (IQR)	6 (5–8)	7 (6–8)	0.162
Follow-up Number of LSA branches, median (IQR)	5 (4–7)	6 (5.5-7)	0.026
LSA branches change, median (IQR)	-1 (-1.5-0)	0 (-1-0)	0.229
Baseline total length of LSAs, mm	112.74 ± 34.88	115.03 ± 34.07	0.774
Follow-up total length of LSAs, mm	94.30 ± 32.23	113.82 ± 31.68	0.010
LSA total length change, mm	-18.44 ± 16.56	-1.21 ± 23.73	0.001
Baseline total distance of LSAs, mm	89.05 ± 30.20	96.811 ± 29.25	0.260
Follow-up total distance of LSAs, mm	78.77 ± 27.23	95.04 ± 29.39	0.014
LSA total distance change, mm	-10.28 ± 15.53	-1.77 ± 19.61	0.039

Except where indicated, data are mean ± SD.

Abbreviations: IQR = interquartile range; LSA = lenticulostriate artery; RSSIs = recent single subcortical infarctions; SD = standard deviation

### Predictors of the Presence of Hemorrhagic foci Determined by Multivariate Analysis

Logistic regression analyses were performed to identify factors associated with the presence of hemorrhagic foci in the LSA territory (Table 3). Due to a significant correlation between the change in LSA total length and distance, we incorporated these two metrics separately in the prediction model as model 1 and model 2, respectively. In model 1, larger DWI lesion volume (OR 1.80, 95% CI 1.13–2.87; P = 0.014) and greater reduction in LSA total length (OR 0.59, 95% CI 0.36–0.96; P = 0.035) were significantly associated with hemorrhagic foci formation, whereas there was no difference in onset to follow-up MRI time (P = 0.124), LSA stem number change (P = 0.621), or baseline NIHSS (P = 0.864) between the two groups. In model 2, only a larger initial lesion volume remained as an independent predictor of the presence of hemorrhagic foci (OR 1.87, 95% CI 1.19–2.95; P = 0.007).

**Table 3:** Multivariable logistic regression analysis for the presence of hemorrhagic foci in RSSIs in the LSA

Variables ^a^	OR (95% CI)	*p* Value
**Model 1**		
Onset to follow-up MRI time, days	1.00 (0.99–1.01)	0.124
DWI lesion volume, median, mm^3^	1.80 (1.13–2.87)	0.014
Baseline NIHSS	1.02 (0.80–1.31)	0.864
LSA total length change, mm	0.59 (0.36–0.96)	0.035
LSA stems change	1.36 (0.40–4.55)	0.621
**Model 2**		
Onset to follow-up MRI time, days	1.00 (0.99–1.01)	0.142
DWI lesion volume, median, mm^3^	1.87 (1.19–2.95)	0.007
Baseline NIHSS	1.03 (0.81–1.31)	0.797
LSA total distance change, mm	1.11 (0.58–2.12)	0.754
LSA stems change	0.38 (0.09–1.58)	0.182

^a^ As LSA total length change was significantly correlated with LSA total distance change, we incorporated them separately into the prediction model (model 1 and model 2)

Abbreviations: CI = confidence interval; DWI = diffusion-weighted imaging; LSA = lenticulostriate artery; NIHSS = National Institutes of Health Stroke Scale; OR = odds ratio

## Discussion

In this study, we have identified a previously underappreciated feature of residual hemorrhagic foci within the index infarct lesion on follow-up SWI in patients with RSSI. Remarkably, nearly half of the patients with RSSI displayed hemorrhagic foci on follow-up MRI scans at a median of 425 days after the stroke onset. Our findings also found that a longer time interval between stroke onset and follow-up imaging, larger infarct size, and anterior circulation location were associated with the formation of hemorrhagic foci. Moreover, patients with hemorrhagic foci were more prone to cavitation and exhibited poorer clinical outcomes during follow-up versus those without hemorrhagic foci. Nonetheless, in the subgroup of RSSIs within the LSA territory, only a larger DWI lesion volume and greater reduction in LSA total length remained independent predictors of the presence of hemorrhagic foci.

Hemorrhagic foci are characterized by a marked hypointense rim surrounding the index lacune (hemosiderin rim; Fig. 2A), occasionally presenting as small, patch-like low signal within the lesion on follow-up SWI (hemosiderin smudge; Fig. 2B). These features are attributed to the paramagnetic effect induced by hemosiderin deposits [23]. It is important to note that other paramagnetic substances in the brain can also produce a dark signal on SWI, including hemorrhage, iron deposition, or deoxygenated hemoglobin trapped in blood clots. Subtle, well-defined hypointense signals along the presumed path of perforating arteries have been reported in RSSIs using T2* gradient echo (GRE) imaging [24, 25]. These signals indicate the presence of deoxygenated hemoglobin trapped in clots, leading to the occlusion of small perforators, a phenomenon termed the susceptibility vessel sign (SVS). However, this acute thrombus sign is unlikely in the chronic stage of RSSIs. Of note, we observed a more substantial decrease in LSA total length in RSSIs, which independently predicted the formation of hemorrhagic foci, suggesting a previous thrombotic occlusion of perforating arteries. Erythrocyte extravasation from a thrombosed “leaky” perforating artery, due to endothelial dysfunction and blood-brain barrier leakage, [24, 26] may eventually lead to hemosiderin deposits, thus resulting in hypointense foci on follow-up SWI.

Additionally, deoxygenated blood in small veins can lead to a hypointense signal on the SWI sequence. One recent study suggested that grouped low-signal small tubular-like structures in the WMH on SWI might represent clusters of small dilated vessels. These vascular clusters were found to be related to damaged tissue showing different grades of cavitation in WMH [27]. However, based on our SWI images, we can clearly identify focal hypointensities within the lacunes as petechial foci, often exhibiting a rim-like distribution at the cavity edges. These radiological features suggest that these focal hypointense foci are unlikely to be over-dilated deep venules. As a result, we posit that these focal hypointense foci in the lacunes after the index RSSI is indicative of hemorrhagic remnants, specifically hemosiderin, as part of the natural progression of RSSIs [28]. Hence, we hypothesize that some of the observed vessel-clusters on SWI in the aforementioned study could be hemosiderin deposits in the WMH, particularly in cases where complete cavitation has formed within the WMH.

Scant radiologic literature exists describing the imaging features of hemorrhagic residues in the evolution of RSSIs. One recent study that echoes the results of our study was conducted using patients with RSSIs in the PICASSO (Prevention of Cardiovascular Events in Ischemic Stroke Patients with High Risk of Cerebral Hemorrhage) trial. Here the authors found that a longer time interval, anterior circulation location, and larger lesion size were associated with the presence of hemorrhagic foci in RSSIs during long-term follow-up, whereas the CSVD markers, such as CMBs, were not different between patients with and without hemorrhagic foci [29]. Surprisingly, the prevalence of hemorrhagic foci (10.4%) in this study was substantially lower than in our study (44.6%), notwithstanding that the PICASSO study population consisted of RSSIs with a history of intracerebral hemorrhage or multiple CMBs, presenting a high hemorrhagic risk. In contrast, we observed a significant association between the formation of lacunes and the presence of hemorrhagic foci. After adjusting for covariates, we found that a greater reduction in LSA total length and larger DWI lesion volume independently predicted the formation of hemorrhagic foci in the LSA territory. The greater reduction in LSA length indicates a more substantial ischemic insult, resulting in a larger infarct within the LSA territory, which, in turn, may contribute to the development of hemorrhagic foci during follow-up. Thus, our findings suggest that the presence of hemorrhagic residues may be related to the degree of ischemia in the initial infarct.

This discrepancy in prevalence could be attributed to the PICASSO study’s utilization of a relatively less sensitive GRE technique for detecting hemorrhagic products [30]. In contrast, we included patients with a larger size cutoff than typically used for RSSIs. Additionally, we strictly adhered to inclusion criteria that focused on enrolling intrinsic CSVD-related RSSIs, deliberately excluding patients with relevant large vessel disease. Because abnormal blood-brain barrier permeability plays a pivotal role in CSVD pathogenesis, [26] this raises the question of whether intrinsic CSVD-related RSSIs exhibit a higher proportion of hemorrhagic remnants compared to those related to large-artery atherosclerosis, a topic warranting further investigation.

Lacunar cavities filled with hemosiderin-laden macrophages are a well-documented feature in pathological investigations [9, 10, 28, 31]. In Fisher’s original observations, “segmental arteriolar disorganization” was identified in 40 out of 50 lacunes with occluded perforating arteries. Among these 40 lesions, 26 (65%) showed evidence of extensive hemorrhagic extravasation through the damaged arterial wall, characterized by the presence of hemosiderin-filled macrophages nearby [9]. The prevalence of hemorrhagic foci in our radiological study appears to align with Fisher’s original pathological description.

The correlation of SWI-identified hypointensities with tissue pathology was investigated in a postmortem MRI study. Several sites were found to be small lacunes ringed by hemosiderin, and scarred vessels were noted to cross nearly all of the cavitary lesions [28]. Another radio-pathological study also identified a significant association between white matter and basal ganglia focal hemosiderin deposits and lacunes in any brain region, supporting an ischemic hypothesis for the origin of hemosiderin/CMB deposits [31]. Extravasated erythrocytes and hemosiderin from ischemic damaged parenchymal and arteriolar walls may migrate through enlarged PVS, propagating an inflammatory reaction along the local microvasculature, which may contribute to the formation of lacunes. These pathological findings indicate a potential association between MRI-detected hemosiderin/CMBs and primarily ischemic process [31, 32]. Thus, the presence of hemorrhagic foci during follow-up after RSSI should not dictate the decision-making of antithrombotic therapy.

The main strength of our study lies in the use of a well-defined cohort, allowing us to determine longitudinal morphological changes after a symptomatic RSSI with standardized imaging acquisition and processing. This approach enables precise identification of the lesion onset, characterization of its evolution, and identification of relevant imaging features.

One limitation of this study, however, is our modest sample size, which might potentially limit the explanatory power of multivariable analyses. This is due to our strict inclusion criteria for RSSIs, excluding patients with competing vascular etiologies, such as large vessel disease or potential sources of cardioembolism. To our knowledge, this study appears to be the first to focus exclusively on hemorrhagic evolution in such a well-defined RSSI cohort. Another limitation is that the interval of follow-up MRI from stroke onset varied significantly between patients, ranging from 321 to 863 days, due to nonconsecutive follow-ups caused by the COVID-19 pandemic. Nonetheless, previous studies have shown that lesion evolution of RSSIs stabilizes after three months [4]. Furthermore, after controlling for the time interval in the multivariate analysis, the interval from the acute stroke did not influence the presence of hemorrhagic foci on follow-up imaging. Lastly, we did not find any association between CSVD markers and hemorrhagic foci formation, largely due to the relatively lower total CSVD burden in our first-ever RSSI cohort. This finding requires confirmation in longitudinal and larger cohorts comprising patients with moderate-to-severe CSVD.

## Conclusions

In summary, hemorrhagic foci, represented as hemosiderin deposits, may be observed in nearly half of the patients with RSSIs on long-term follow-up MRI. Larger lesion sizes and greater reduction in LSA total length were independently associated with hemorrhagic foci formation within the index RSSIs in the LSA territory. Hemorrhagic foci within the lacunes might represent hemosiderin residues from previously occluded perforating arteries. Larger longitudinal studies are necessary to confirm this hypothesis and to unravel the possible clinical and prognostic value for patients with RSSI.

## Electronic Supplementary Material

Below is the link to the electronic supplementary material.

Supplementary Material 1

## Data Availability

The data from this study are available from the corresponding author upon reasonable request.
