# Supplementary material for: Prevalence and Predictors of Hemorrhagic Foci on Long-term Follow-up MRI of Recent Single Subcortical Infarcts
Source: Transl Stroke Res. 2023 Dec 14;16(2):410–20. doi: 10.1007/s12975-023-01224-7 (PMC11976363; doi:10.1007/s12975-023-01224-7)
Supplement: Supplementary file 1 — Supplementary Material 1 [file 12975_2023_1224_MOESM1_ESM.pdf]

Prevalence and Predictors of Hemorrhagic Foci on Long-term Follow-up MRI of Recent Single Subcortical Infarcts

Shuai Jiang, PhD,[1 *] Wen-Zuo Shang, MD,[1, *] Jing-Yu Cui, MD,[1] Yu-Ying Yan, PhD,[1] Tang Yang, MD,[1] Yi Hu, MD,[1] Le Cao, MD,[1] Xun Yue MD,[2] Ruo-Su Pan, MD,[1] Chen Ye, PhD,[1] Jia-Yu Sun, PhD,[2, #] Bo Wu, PhD[1, #]

[1] Department of Neurology, West China Hospital, Sichuan University, Chengdu, China

[2] Department of Radiology, West China Hospital, Sichuan University, Chengdu, China

[#]*Correspondence to*

Bo Wu, Department of Neurology, West China Hospital, Sichuan University, No. 37, Guo Xue Xiang, Chengdu 610041, China. Email: dr.bowu@hotmail.com or Jiayu Sun, Department of Radiology, West China Hospital, Sichuan University, Guo Xue Xiang 37, Chengdu 610041, China. Email: sjy080512@163.com.

*These authors contributed equally to the manuscript.

SUPPLEMENTAL MATERIALS

Table S1 Sequence parameters of multimodal brain MRI scans

**Sequence**	**T1WI**	**T2WI**	**FLAIR**	**DWI**	**SWI**	**VWI**	**TOF-MRA**
TR (ms)	1600	4000	9000	4000	28	900	22
TE (ms)	9.2	93	93	91	20	14	4
FOV (mm)	220×176	220×184	220×196	220×220	230×180	170×170	170×170
Matrix	320×208	320×288	256×223	192×192	384×384	320×320	320×320
Slice thickness (mm)	5	5	5	5	2	0.53	0.53
Slice gap (mm)	1.5	1.5	1.5	1.5	0.4	0	0
NO. slices	21	21	21	21	72	240	155
Acquisition time (mm: ss)	1:41	0:58	1:32	1:50	6:02	8:10	6:08

Abbreviations: DWI = diffusion-weighted imaging; FLAIR = fluid attenuation inversion recovery; FOV = field of view; MRA = magnetic resonance angiography; SWI = susceptibility weighted imaging; TE = echo time; TR = repetition time; TOF = Time-of-flight; T1WI = T1-weighted imaging; T2WI = T2-weighted imaging; VWI = vessel wall imaging.

Table S2 Comparison of demographics, clinical data and MRI findings between RSSIs in the LSA territory with versus without hemorrhagic foci

Characteristics	Hemorrhagic foci (n=40)	No hemorrhagic foci (n=36)	*p* Value
**Demographics**			
Male, n (%)	36 (90.0)	30 (83.3)	0.503
Age, y (mean ± SD)	53.93±10.07	52.58±10.27	0.567
**Risk factors, n (%)**			
Hypertension	20 (50.0)	24 (66.7)	0.165
Diabetes	16 (40.0)	9 (25.0)	0.209
Hyperlipidemia	17 (42.5)	10 (27.8)	0.181
Current smoking	18 (45.0)	22 (61.1)	0.160
**Clinical data, median (IQR)**			
Baseline NIHSS	4 (2–8)	2 (1–4)	0.020
Baseline mRS	3 (1.5–4)	2 (1–3)	0.011
Follow-up mRS	1.5 (1-2)	1 (0-1)	0.015
Follow-up mRS ≥ 2, n (%)	20 (50.0)	7 (19.4)	0.005
Onset to baseline MRI time	5 (4-7)	6 (3–13)	0.208
Onset to follow-up MRI time	481.5 (407.5-665.5)	436 (385.5-520)	0.043
**Medication, n (%)**			
Dual antiplatelet therapy	22 (55.0)	17 (47.2)	0.498
**Infarct Dimensions, median (IQR)**			
DWI lesion axial diameter, mm	19.2 (16.1-26.1)	12.8 (10.1-17.9)	<0.001
DWI lesion volume, cm^3	2.84 (1.53-6.27)	0.73 (0.39-2.12)	<0.001
Follow-up T1 lacune axial diameter [a], mm	9.75 (8.18-12.30)	7.80 (5.75-10.40)	0.008
Follow-up T1 lacune volume [a], cm^3	0.35 (0.14-0.62)	0.14 (0.06-0.22)	0.001
**RSSI evolution, n (%)**			0.046
Cavitation	36 (90.0)	26 (72.2)	
No cavitation	4 (10.0)	10 (27.8)	
**CSVD markers, n (%)**			
Extensive perivascular WMH (Fazekas 3)	2 (5.0)	7 (19.4)	0.112
Extensive deep WMH (Fazekas 2–3)	2 (5.0)	6 (16.7)	0.140
Moderate–extensive basal ganglia PVS	19 (47.5)	17 (47.2)	0.981
Lacunes (≥l)	15 (37.5)	18 (50.0)	0.272
Cerebral microbleeds(≥l)	19 (47.5)	17 (47.2)	0.981

Data are presented as mean ±SD, median (IQR) or number (%).

[a] Available in 62 patients, 12 lesions evolved into WMH and 2 lesions that disappeared on follow-up imaging were excluded.

Abbreviations: BG-PVS = basal ganglia perivascular spaces; CSVD= cerebral small vessel disease; DWI = diffusion-weighted imaging; mRS = modified Rankin Scale; NIHSS = National Institutes of Health Stroke Scale; IQR = interquartile range; RSSIs, recent single subcortical infarctions; SD = standard deviation; T1 = T1 weighted image; WMH = white matter hyperintensities.
